# Enhancing genomic prediction for key production traits in chickens through ultrasound phenotyping and multi-model comparative analysis

**DOI:** 10.1186/s40104-026-01384-0

**Published:** 2026-04-25

**Authors:** Ranran Zhu, Yuxiang Jiang, Wanyi Xiong, Yu Zhang, Ziyi Lian, Danni Gou, Zhandeng Li, Xiuping Wang, Xuemei Deng

**Affiliations:** 1 Sanya Institute, China Agricultural University, Sanya, 572025 China; 2 State Key Laboratory of Animal Biotech Breeding, Beijing Key Laboratory for Animal Genetic Improvement and Key Laboratory of Animal Genetics, Breeding and Reproduction of the Ministry of Agriculture, College of Animal Science and Technology, China Agricultural University, Beijing, 100193 China; 3 Hainan (Tanniu) Wenchang Chicken Co., Ltd., Haikou, 570100 China

**Keywords:** Genomic prediction, Machine learning, Ultrasound phenotyping, Wenchang chicken, Whole-genome sequencing

## Abstract

**Background:**

Growth performance and carcass traits are economically vital in poultry breeding. In Wenchang chickens, reducing excessive abdominal fat represents a critical breeding objective. However, as a typical carcass trait, abdominal fat thickness has traditionally been measurable only post-slaughter, resulting in inefficient and costly selection processes that hinder genetic progress for these traits. To overcome this limitation, we developed an integrated approach combining non-invasive ultrasound phenotyping and multi-model genomic selection to evaluate growth and fat-related traits in Wenchang chickens.

**Results:**

We genotyped 3,737 chickens using the “Jingxin No.1” 55K SNP array and performed longitudinal measurement of abdominal fat thickness (AFT) via ultrasound imaging. A comprehensive evaluation of genomic prediction models revealed that WGBLUP (informed by wssGWAS), and GBLUP models based on LD-pruned whole-genome sequencing (WGS) data significantly outperformed standard GBLUP, with accuracy gains of 5.25% and 6.58%–15.30%, respectively. Among the machine learning algorithms tested, kernel ridge regression (KRR) and support vector regression (SVR) achieved the highest predictive improvement (3.00%–4.15%) while maintaining superior computational efficiency, whereas ensemble methods provide no consistent advantage.

**Conclusions:**

Our work established ultrasound imaging as a scalable, non-invasive phenotyping platform for poultry breeding. Results demonstrated that integrating wssGWAS-derived biological priors with WGS data substantially improves genomic prediction accuracy for complex traits. This integration, enhanced by computationally efficient machine learning algorithms, provides a powerful and practical strategy to accelerate genetic gain.

**Supplementary Information:**

The online version contains supplementary material available at 10.1186/s40104-026-01384-0.

## Background

Genomic selection (GS) [1] has become a cornerstone of modern animal and plant breeding, enabling the prediction of genetic values for complex traits using genome-wide markers [2–4]. By leveraging reference populations with both genotypic and phenotypic data, GS facilitates the selection of superior candidates, significantly accelerating genetic gain [5]. The accuracy of genomic prediction, however, is influenced by multiple factors, including trait heritability, reference population size, marker density, and statistical methods employed [6, 7].

The evolution of statistical methodologies has been central to advancing GS. Standard approaches such as genomic best linear unbiased prediction (GBLUP) assume an infinitesimal genetic architecture [8]. To better capture trait-specific genetic architectures, more sophisticated methods have been developed. These include weighted GBLUP (WGBLUP); genomic feature BLUP (GFBLUP), which focuses on functionally informed marker subsets; and various Bayesian methods that allow for flexible prior distributions of marker effects [9–11]. More recently, machine learning (ML) algorithms such as kernel ridge regression (KRR) and random forest (RF) have been introduced to GS, offering the potential to model complex non-linear relationships without pre-specified genetic assumptions [9, 12].

Evidence from previous studies underscores the context-dependent nature of these methods. In chicken, Romé et al. [13] demonstrated that WGBLUP improved the prediction accuracy for body weight traits by 2%–7% over GBLUP, and further gains were achieved by including top GWAS hits as fixed effects. Similarly, Li et al. [14] reported that ML methods outperformed traditional methods for certain traits in laying hens, with integration of GWAS prior boosting accuracy by up to 27%. In yellow-feathered broilers, Yang et al. [15] showed that incorporating significant QTLs into GFBLUP increased prediction accuracy for body weights by over 13%. Despite these advances, the performance of prediction models remains highly variable across traits and populations, particularly for complex characteristics like fat deposition. This inconsistency highlights a critical challenge: optimal GS strategy appears to be contingent on the underlying genetic architecture of the trait, yet a systematic framework for matching models to traits is still lacking.

Conventional measurement of carcass traits such as abdominal fat thickness relies on post-slaughter assessment, which is not only time-consuming and costly but also precludes selection in live animals. To accelerate breeding progress, researchers have developed various in vivo measurement techniques for predicting carcass traits. In poultry research, ultrasound scanning has been successfully combined with live body measurements to predict abdominal fat content [16, 17] as well as other key carcass traits, such as breast muscle weight [18] and skeletal characteristics [19]. This methodological approach has also been effectively extended to other livestock species, including body fat prediction in Pekin ducks [20] and carcass chemical composition assessment in sheep [21]. These studies demonstrate that B-mode ultrasonography combined with live measurement provides a reliable and efficient means for the in vivo evaluation of economically important traits in poultry and livestock. Building on this technical foundation, this study aims to apply ultrasound-based in vivo detection to measure abdominal fat thickness in Wenchang chickens, with the objectives of evaluating its potential application in the breeding of this indigenous breed and supplying accurate phenotypic data for the establishment of an efficient genomic selection program.

To address this gap and to overcome the high costs associated with phenotyping carcass traits, we conducted a comprehensive genomic analysis in Wenchang chickens. We genotyped 3,737 individuals from two consecutive generations using the “Jingxin No.1” 55K SNP chip, followed by genotype imputation to whole-genome sequencing (WGS) density. A key innovation of our study is the integration of a novel, ultrasound-derived phenotype for abdominal fat thickness (AFT)—a trait typically measured postmortem—with a multi-faceted GS evaluation. We systematically compared the performance of: (1) conventional linear mixed models (GBLUP, ssGBLUP); (2) a prior-informed model (WGBLUP) incorporating weights from weighted single-step GWAS (wssGWAS); (3) five ML algorithms (KRR, SVR, RF, LightGBM, and AdaBoost.R2) optimized via grid search; and (4) GBLUP models based on GBLUP LD-pruned WGS data to assess the impact of marker density.

Through rigorous 10-replicates, five-fold cross-validation (CV), this study aims to: (1) assess whether integrating GWAS-derived prior information with ML algorithms enhances predictive performance; (2) compare the predictive efficiency of conventional, prior-informed, and machine learning models; and (3) evaluate the impact of marker density by comparing SNP chip data with imputed WGS data under varying LD pruning thresholds.

By systematically comparing these diverse methodologies, this study establishes a trait-informed decision framework to guide breeders in selecting optimal genomic prediction strategies, thereby enhancing both the efficiency and accuracy of breeding programs for economically important traits in poultry.

## Materials and methods

### Experiment animals, phenotypes and pedigree

A total of 3,737 M-line hens were sourced from the Hainan Wenchang Chicken Breeding Farm, including 2,304 from the 19^th^ generation and 1,433 from the 20^th^ generation, all reared under uniform environmental conditions. Phenotypic traits were recorded at three developmental stages (22, 32, and 45 weeks of age), including body weight (BW) and a novel ultrasound-derived trait—AFT. As a result of culling and slaughtering during the rearing process, the number of sampled birds decreased with age, leading to missing phenotypic records for some individuals. Prior to statistical analysis, quality control procedures and normality tests were rigorously performed. Descriptive statistics of the phenotypic data are presented in Table 1.

**Table 1:** Descriptive statistics and genetic parameter estimates of traits

Traits^a^	22w-BW	32w-BW	45w-BW	22w-AFT	32w-AFT	45w-AFT
Ne^b^	3,434	3,153	2,297	2,365	2,628	1,630
Max	2.372	2.894	2.972	1.298	1.858	1.913
Min	1.062	1.225	1.413	0.197	0.197	0.199
Mean	1.774	1.905	1.969	0.750	0.698	0.762
Std	0.172	0.228	0.276	0.210	0.234	0.213
σ_*g*_^2^	0.004	0.013	0.014	0.007	0.013	0.010
σ_e_^2^	0.022	0.036	0.057	0.032	0.038	0.048
h^2^(SE)^c^	0.155(0.005)	0.264(0.007)	0.200(0.008)	0.175(0.007)	0.254(0.008)	0.174(0.009)

^a^22w-AFT, 32w-AFT, 45w-AFT, 22w-BW, 32w-BW, and 45w-BW represent abdominal fat thickness (ultrasound-derived novel traits) and body weight measured at 22, 32, and 45 weeks of age in the experimental population

^b^Ne: Effective number of records for each trait

^c^h^2^(SE): Heritability estimates of traits with standard errors in parentheses

AFT was quantitatively measured using a DCU10 digital color Doppler ultrasound system (Xuzhou Kaixin Electronic Instrument Co., Ltd., China) equipped with a 7.5-MHz linear-array probe operating in B-mode grayscale imaging. Prior to scanning, chickens were gently removed from their cages and manually restrained in a dorsal recumbent position on the operator’s left leg, after which feathers in the abdominal region were removed. An appropriate amount of medical coupling gel was applied to the probe surface to ensure effective acoustic transmission. During ultrasonography, the probe was positioned 3–5 cm posterior to the keel bone with an imaging depth of 5.5 cm. All instrument parameters were maintained within the safety range (thermal index, TIS = 0.2; mechanical index, MI = 0.8), ensuring no adverse effects on the examined birds. Considering the heterogeneity of abdominal fat distribution, three independent measurements were taken from the left, middle, and right abdominal regions. The saved ultrasound images were later analyzed using Image-Pro Plus 6.0 software, where clear upper and lower boundaries of the fat layer were manually demarcated for measurement. The mean value of these triplicate measurements was calculated and used as the final AFT phenotype for each individual.

Initial parent–offspring assignments from farm records were verified against genotype data, leading to the correction of 246 erroneous assignments using genotype information; a further 54 pairs were annotated as having unknown parental status in the final pedigree file, which included 4,472 individuals from generations 18 to 20. All phenotypic measurements were conducted on-site by trained personnel to ensure data consistency and measurement accuracy.

### Genotyping by SNP chip and genotype imputation

Genomic DNA was extracted from whole blood samples of 3,737 Wenchang chickens collected via wing vein puncture, and genotyping was conducted using the Jingxin 55K SNP chip, with SNP positions mapped to the GRCg6a reference genome. Quality control was conducted using PLINK v1.90b6.21 [22], which included the following steps: (1) SNPs with a call rate of less than 98% were removed; (2) SNPs with a minor allele frequency (MAF) of less than 0.05 were excluded; (3) SNPs significantly deviating from Hardy–Weinberg equilibrium (*P* < 10^–6^) were eliminated; (4) individuals with a genotype call rate of less than 95% were excluded; and (5) SNPs located on chromosomes with unknown physical positions were removed. A total of 41,086 high-quality SNPs from 3,737 individuals were retained for subsequent genetic evaluation and wssGWAS analysis.

To further enhance genomic resolution, 312 Wenchang chickens closely related to the chip-genotyped population were selected for whole‑genome sequencing. Sequencing libraries were constructed from high‑quality DNA (fragmented to 300–500 bp) and sequenced on the DNBSEQ‑T7 platform (MGI) to an average depth of 15 ×. After alignment to the GRCg7b reference genome using BWA, the resulting data served as a reference panel for genotype imputation. Throughout the process, stringent quality control was applied, including assessments of DNA concentration and integrity, library preparation monitoring, and post‑sequencing QC with FastQC and Samtools, to ensure data reliability. Reference-based imputation was conducted using Beagle version 5.4 [23] on the 55K SNP chip data. Only SNPs with consistent positions between the chip and sequencing datasets and a post-imputation dosage R-squared (*R*^2^) ≥ 0.95 were retained. The imputed data were then subjected to quality control using the same criteria as the chip data, resulting in 6,360,109 high-quality SNPs for downstream analysis. Subsequently, LD pruning was performed using PLINK v1.90b6.21 [22] with the command “--indep-pairwise 50 5 < r^2^ > ” under LD thresholds (r^2^) of 0.2, 0.4, and 0.6, yielding 554,660, 1,463,395, and 2,425,353 SNPs, respectively, for subsequent genomic prediction analyses.

### Derivation of corrected phenotypes

To avoid redundant accounting of parental information, the adjusted phenotypic values (*y*_c_) were used as the response variable in genomic prediction. For each trait, estimated breeding values (EBVs) were obtained using pedigree-based ABLUP under a single-trait animal model, where the numerator relationship matrix (***A*** matrix) was constructed from the complete pedigree information to quantify additive genetic relationships among individuals.

1 $${\boldsymbol{y}}={\boldsymbol{X}}{\boldsymbol{b}}+{\gamma}{\boldsymbol{W}}+{\boldsymbol{Z}}{\boldsymbol{a}}+{\boldsymbol{e}}$$

where $${\boldsymbol{y}}$$ represents the phenotypic observations of the traits in Wenchang chickens; $${\boldsymbol{b}}$$ is the vector of fixed effects (e.g., batch, effect); $${\boldsymbol{W}}$$ is a vector of covariate of the phenotypic value (e.g., pc1, pc2, pc3); $$\gamma$$ is the regression coefficient associated with $${\boldsymbol{W}}$$; $${\boldsymbol{a}}$$ is the random additive genetic effect of the individual, assuming a normal distribution: $${\boldsymbol{a}}$$ ~ N($$\boldsymbol{0}, \boldsymbol A$$$${{\sigma }_{a}}^{2}$$), where $${{\sigma }_{a}}^{2}$$ is the additive genetic variance; $${\boldsymbol{e}}$$ is the vector of random residuals with distribution of N ($$\boldsymbol{0}, \boldsymbol {I}$$ $${{\sigma }_{e}}^{2}$$), $${{\upsigma }_{e}}^{2}$$ is the residual variance, $$\boldsymbol I$$ is the identity matrix. $${\boldsymbol{X}}$$ and $${\boldsymbol{Z}}$$ are incidence matrices associating $${\boldsymbol{b}}$$ and $${\boldsymbol{a}}$$ with $${\boldsymbol{y}}$$, respectively. EBVs were calculated using the blupf90 program within the BLUPF90 software suite [24]. *y*_c_ were obtained by adding the estimated residuals to the corresponding EBVs [25].

### Genomic prediction models

In this study, multiple genomic prediction approaches were employed to estimate genomic estimated breeding values (GEBVs) for target traits. These included three conventional models—GBLUP, ssGBLUP, and WGBLUP—as well as five machine learning regression algorithms: KRR, SVR, RF, LightGBM, and AdaBoost.R2. To assess the impact of SNP marker density on prediction accuracy, SNP chip data were first imputed to WGS level. LD-based pruning was then applied to generate SNP subsets under three LD thresholds (LD = 0.2, 0.4, and 0.6). GBLUP models were constructed for each SNP subset to perform prediction analyses. All models were evaluated using five-fold cross-validation repeated ten times to ensure robustness and stability of the prediction performance. The GBLUP, ssGBLUP, and WGBLUP models were implemented using the blupf90 software package [24].

### Genomic best linear unbiased prediction

For genomic prediction, we first employed the standard genomic best linear unbiased prediction model. This model estimates individual genomic breeding values by constructing a genomic relationship matrix ($\boldsymbol G$-matrix) based on genome-wide markers, which replaces the traditional pedigree-based relationship matrix.

2 $${{\boldsymbol{y}}}_{{\boldsymbol{c}}}=\boldsymbol{\mu }+{\boldsymbol{Z}}{\boldsymbol{g}}+{\boldsymbol{e}}$$

where ${{\boldsymbol{y}}}_{{\boldsymbol{c}}}$ is the vector of adjusted phenotypic values, ${\boldsymbol{\mu}}$ is the overall mean, ${\boldsymbol{Z}}$ is the incidence matrix relating observations to individuals, ${\boldsymbol{g}}$ is the vector of additive genetic values assumed to follow a normal distribution ${\boldsymbol{g}}$ ~ N ($\boldsymbol{0}, \boldsymbol{G}$ ${{\sigma }_{g}}^{2}$), and ${\boldsymbol{e}}$ is the vector of random residuals with ${\boldsymbol{e}}$ ~ N (**0**, I ${{\sigma }_{e}}^{2}$). Here, $\boldsymbol G$ denotes the genomic relationship matrix ($\boldsymbol G$ matrix), while ${{\sigma }_{g}}^{2}$ and ${{\sigma }_{e}}^{2}$ represent the additive genetic variance and residual variance, respectively.

### Single-step genomic best linear unbiased prediction

Unlike GBLUP, the ssGBLUP model constructs a unified ${\boldsymbol{H}}$ matrix by integrating the pedigree-based relationship matrix ($\boldsymbol A$ matrix) with the genomic relationship matrix ($\boldsymbol G$ matrix) [26]. It was assumed that ${\boldsymbol{g}}$ followed a normal distribution N ($\boldsymbol{0}, \boldsymbol {H}$ ${{\sigma }_{g}}^{2}$). The inverse of matrix $\boldsymbol H$ was:

3 $${{\boldsymbol{H}}}^{-1}=\left[\begin{array}{cc}0& 0\\ 0& {{{\boldsymbol{G}}}_{{\boldsymbol{w}}}}^{-1}-{{{\boldsymbol{A}}}_{22}}^{-1}\end{array}\right]+{{\boldsymbol{A}}}^{-1}$$

where ${\boldsymbol{A}}$ is the numerator relationship matrix based on pedigree for all animals, ${{{\boldsymbol{A}}}_{22}}^{-1}$ is the inverse of the pedigree-based relationship matrix for the genotyped animals, and ${{{\boldsymbol{G}}}_{{\boldsymbol{w}}}}^{-1}$ is the inverse of the genomic relationship matrix, ${{\boldsymbol{G}}}_{{\boldsymbol{w}}}=0.95{\boldsymbol{G}}+0.05{\mathbf{A}}_{22}$.

### Weighted genomic best linear unbiased prediction

The WGBLUP model is conceptually similar to the conventional GBLUP model, with the key distinction that it incorporates marker-specific weights when constructing the genomic relationship matrix. In WGBLUP, all SNPs are assigned weights, which are iteratively updated based on their estimated contributions to the trait of interest. The formulas below outline the computational procedures for deriving weighted SNPs through the WSSGWAS approach:

- Step 1: let $${t=0,{\boldsymbol{D}}}_{\left(t\right)}={\boldsymbol{I}},{\mathsf{G}}_{\left(t\right)}={\boldsymbol{Z}}{{\boldsymbol{D}}}_{\left(t\right)}{{\boldsymbol{Z}}}^{\prime}\lambda,\ \text{and }\lambda ={\sum }_{i=1}^{M}2{p}_{i}\left(1-{p}_{i}\right)$$;
- Step 2: Compute ${\widehat{{\boldsymbol{a}}}}_{{\boldsymbol{g}}}$ by ssGBLUP;
- Step 3: Calculate $${\widehat{{\boldsymbol{u}}}}_{\left(t\right)}=\uplambda {{\boldsymbol{D}}}_{\left(t\right)} \mathbf{Z}\mathbf{^{\prime}}{{{\boldsymbol{G}}}_{\left(t\right)}}^{-1}{\widehat{{\boldsymbol{a}}}}_{{\boldsymbol{g}}}$$;
- Step 4: Calculate ${{d}_{i_{(t+1)}}^{*}} ={{\widehat{u}}_{i_{\left(t\right)}}^{2}}{2p}_{i}(1-{p}_{i})$ for all SNPs;
- Step 5: Normalize $${\mathbf{D}}_{\left(t+1\right)}=\frac{\mathrm{tr}({\mathbf{D}}_{\left(0\right)})}{\mathrm{tr}({{\mathbf{D}}^{\bullet }}_{\left(t+1\right)})}{{\mathbf{D}}^{\bullet }}_{\left(t+1\right)}$$;
- Step 6: Calculate ${\boldsymbol{G}}$ for the next iteration, ${\boldsymbol{G}}_{\left(t+1\right)}={\boldsymbol{Z}}{{\boldsymbol{D}}}_{\left(t+1\right)}{\boldsymbol{Z}}\boldsymbol{^{\prime}}\lambda$;
- Step 7: let $$t=t+1$$;
- Step 8: Loop to step 2 or 3.

As suggested by Wang et al. [27], the procedure was run for two iterations. Each non-overlapping window included 20 consecutive SNPs, and the genetic variances explained by these windows were calculated following the method of Wang et al. [27].

4 $${\boldsymbol{G}}=\frac{{\boldsymbol{Z}}{\boldsymbol{D}}{{\boldsymbol{Z}}}^{\boldsymbol{^{\prime}}}}{{\sum }_{i=1}^{M}2{p}_{i}\left(1-{p}_{i}\right)}$$

where ${\boldsymbol{Z}}$ is a matrix of gene content adjusted for allele frequencies (); ${\boldsymbol{D}}$ is a diagonal matrix of weights for SNP variances (initially ${\boldsymbol{D}}$ = $\boldsymbol I$); $M$ is 0, 1, or 2 for *aa*, *Aa*, and *AA*, respectively the number of SNPs, and ${p}_{i}$ is the minor allele frequency of the $i$^th^ SNP. This iterative weighting approach allows WGBLUP to prioritize SNPs based on their trait contributions, enhancing both prediction accuracy and model interpretability.

### Kernel ridge regression

KRR, as a nonparametric extension of ridge regression, is effective in capturing complex nonlinear relationships in the data [28]. KRR maps the input data into a high-dimensional kernel space using nonlinear kernel functions, where a linear ridge regression model is then constructed to enable linear separability in this transformed space. The optimal linear function in the kernel space is selected by minimizing the ridge-regularized mean squared error (MSE) loss function. The final predictive model in KRR can be expressed as:

5 $${\boldsymbol{y}}({x}_{i})={{\boldsymbol{k}}\boldsymbol{^{\prime}}({\boldsymbol{K}}+\lambda {\boldsymbol{I}})}^{-1}\widehat{{\boldsymbol{y}}}$$

where $\lambda$ is the regularization parameter, ${\boldsymbol{K}}$ is the Gram matrix defined by ${{\boldsymbol{K}}}_{ij}=\mathrm{K}\left({{\boldsymbol{x}}}_{{\boldsymbol{i}}},{{\boldsymbol{x}}}_{{\boldsymbol{j}}}\right)=\varnothing \left({{\boldsymbol{x}}}_{{\boldsymbol{i}}}\right)*{\varnothing \left({{\boldsymbol{x}}}_{{\boldsymbol{j}}}\right)}^{\mathrm{T}}$, and $\boldsymbol I$ is the identity matrix. For a training dataset with *n* samples, the kernel matrix ${\boldsymbol{K}}$ is defined as:

6 $${\boldsymbol{K}}={\left[\begin{array}{cccc}\mathrm{K}\left({{\boldsymbol{x}}}_{1},{{\boldsymbol{x}}}_{1}\right)& \mathrm{K}\left({{\boldsymbol{x}}}_{1},{{\boldsymbol{x}}}_{2}\right)& \cdots & \mathrm{K}\left({{\boldsymbol{x}}}_{1},{{\boldsymbol{x}}}_{n}\right)\\ \mathrm{K}\left({{\boldsymbol{x}}}_{2},{{\boldsymbol{x}}}_{1}\right)& \mathrm{K}\left({{\boldsymbol{x}}}_{2},{{\boldsymbol{x}}}_{2}\right)& \cdots & \mathrm{K}\left({{\boldsymbol{x}}}_{2},{{\boldsymbol{x}}}_{n}\right)\\ \vdots & \vdots & \vdots & \vdots \\ \mathrm{K}\left({{\boldsymbol{x}}}_{n},{{\boldsymbol{x}}}_{1}\right)& \mathrm{K}\left({{\boldsymbol{x}}}_{n},{{\boldsymbol{x}}}_{2}\right)& \cdots & \mathrm{K}\left({{\boldsymbol{x}}}_{n},{{\boldsymbol{x}}}_{n}\right)\end{array}\right]}_{n\boldsymbol{*}n}$$

In this study, a grid search strategy combined with internal five-fold CV was employed to optimize both the kernel function and the regularization parameter λ, aiming to achieve optimal model performance.

### Support vector regression

Support vector machines (SVM), based on statistical learning theory, are widely applied in both classification and regression tasks. SVR extends SVM to regression problems by using linear or nonlinear kernel functions to map input data into a high-dimensional feature space, where a linear regression model is constructed to fit continuous response variables [29, 30]. The SVR model can be formulated as:

7 $$f(x)={{\beta }_{0}+{\boldsymbol{h}}({\boldsymbol{x}})}^{\mathrm{T}}{\boldsymbol{\beta}}$$

where ${{\boldsymbol{h}}({\boldsymbol{x}})}^{\mathrm{T}}{\boldsymbol{\beta}}$ represents the kernel-induced linear combination, ${\boldsymbol{\beta}}$ is the weight vector, and ${\beta }_{0}$ is the intercept. SVR aims to minimize the following objective function:

8 $$\underset{{\beta}_{0},\beta}{\mathrm{min}}\frac{1}{2}{\Vert{\boldsymbol{\beta}}\Vert}^{2}=C\sum\limits_{i=1}^{n}{V}_{\varepsilon}({y}_{i}-f({{\boldsymbol{x}}}_{{\boldsymbol{i}}}))$$

where ${\mathrm{V}}_{\varepsilon }(r)$ is the ε-insensitive loss function defined as:

9 $${\mathrm{V}}_{\varepsilon }(r)=\left\{\begin{array}{c}0,\ if\left|r\right|<\varepsilon \\ \left|r\right|-\varepsilon,\ otherwise\end{array}\right.$$

The regularization parameter C controls the trade-off between the model's complexity and prediction error. $\Vert \Vert$ denotes the norm in the reproducing kernel Hilbert space. After optimization, the final form of the SVR model can be written as:

10 $$f(x)=\sum\limits_{i=1}^{m}({\widehat{a}}_{i}-{a}_{i})\mathrm{k}({\boldsymbol{x}},{{\boldsymbol{x}}}_{{\boldsymbol{i}}})$$

where $\mathrm{k}({\boldsymbol{x}},{{\boldsymbol{x}}}_{{\boldsymbol{i}}})$ =${\varnothing \left({{\boldsymbol{x}}}_{{\boldsymbol{j}}}\right)}^{\mathrm{T}}\varnothing \left({{\boldsymbol{x}}}_{{\boldsymbol{i}}}\right)$ is the kernel function, and ${\widehat{a}}_{i}$ and ${a}_{i}$ are the Lagrange multipliers obtained from the dual optimization [31]. In this study, an internal fivefold cross-validation strategy combined with grid search was applied to select the optimal kernel function and tune the hyperparameters $C$ and $\gamma$ to enhance model generalization.

### Random forest

RF is an ensemble-based machine learning algorithm that constructs multiple decision trees and integrates their outputs by voting (for classification) or averaging (for regression) to enhance predictive accuracy and reduce overfitting risk [32]. Each tree in the forest is trained on a different random subset of the data, enabling the model to maintain strong generalization ability while reducing variance [31]. The RF regression model is expressed as:

11 $$y=\frac1M\sum_{m=1}^Mt_m(\psi_m(x))$$

where $y$ represents the predicted value, $t_m(\psi_m(\textit{x}))$ denotes the output of the *m*^th^ decision tree, and *M* is the total number of trees in the forest. Each tree processes the input features through a hierarchical structure, and the predicted value is assigned based on the output of the terminal leaf node. Internal fivefold cross-validation was employed in combination with grid search to optimize the number of trees (*M*) and the maximum tree depth.

Genotype vectors (encoded as 0, 1, and 2) were employed as predictor variables, while *y*_c_ served as the response in all five machine learning models. The Scikit-learn package (version 1.6.0) in Python was used for model training and evaluation. Grid search was used to optimize hyperparameters, and the best-performing model within each cross-validation fold was identified based on the highest Pearson correlation between predicted and actual phenotypes.

### Cross-validation and genome prediction effect

To evaluate the accuracy of genomic prediction, a repeated five-fold CV scheme was employed, involving ten repetitions for a total of 50 validation rounds. Prediction accuracy was defined as the Pearson correlation coefficient between *y*_c_ and the corresponding GEBVs within the validation subsets. This metric directly measures the model's predictive performance in the target population. Although standardized accuracy ($r/\mathrm h$) is useful for comparing traits with differing heritabilities, the unstandardized correlation (*r*) is more widely adopted in the literature, enabling direct comparisons with previous studies. In each repetition, the 3,737 individuals were randomly partitioned into five subsets of approximately 747 birds each. Four subsets were used to train the prediction model, while the remaining one served as the validation set.

Prediction bias was assessed by regressing *y*_c_ on GEBVs in the validation population, with the resulting regression coefficient reflecting the degree of inflation or deflation in the genomic predictions. Both prediction accuracy and bias were averaged across all 50 CV rounds to obtain the final estimates.

To further assess the performance of the regression models, MSE and mean absolute error (MAE) were also calculated. MSE incorporates both variance and bias, where a lower value indicates better model fit to the observed data. MAE, on the other hand, offers a more intuitive measure of the average prediction error. These metrics were computed using the following formulas:

12 $$\mathrm{MSE}=\frac{1}{m}{\sum }_{i=1}^{m}{({f}_{i}-{y}_{i})}^{2},\ {and\ MAE}=\frac{1}{m}{\sum }_{i=1}^{m}\left|{f}_{i}-{y}_{i}\right|$$

where *m* represents the number of individuals in the validation set for each CV round, *f* denotes the predicted GEBV vector, and *y* is the vector of *y*_c_. The final MSE and MAE values were averaged across all cross-validation rounds to represent overall model performance.

## Results

**Fig. 1:**
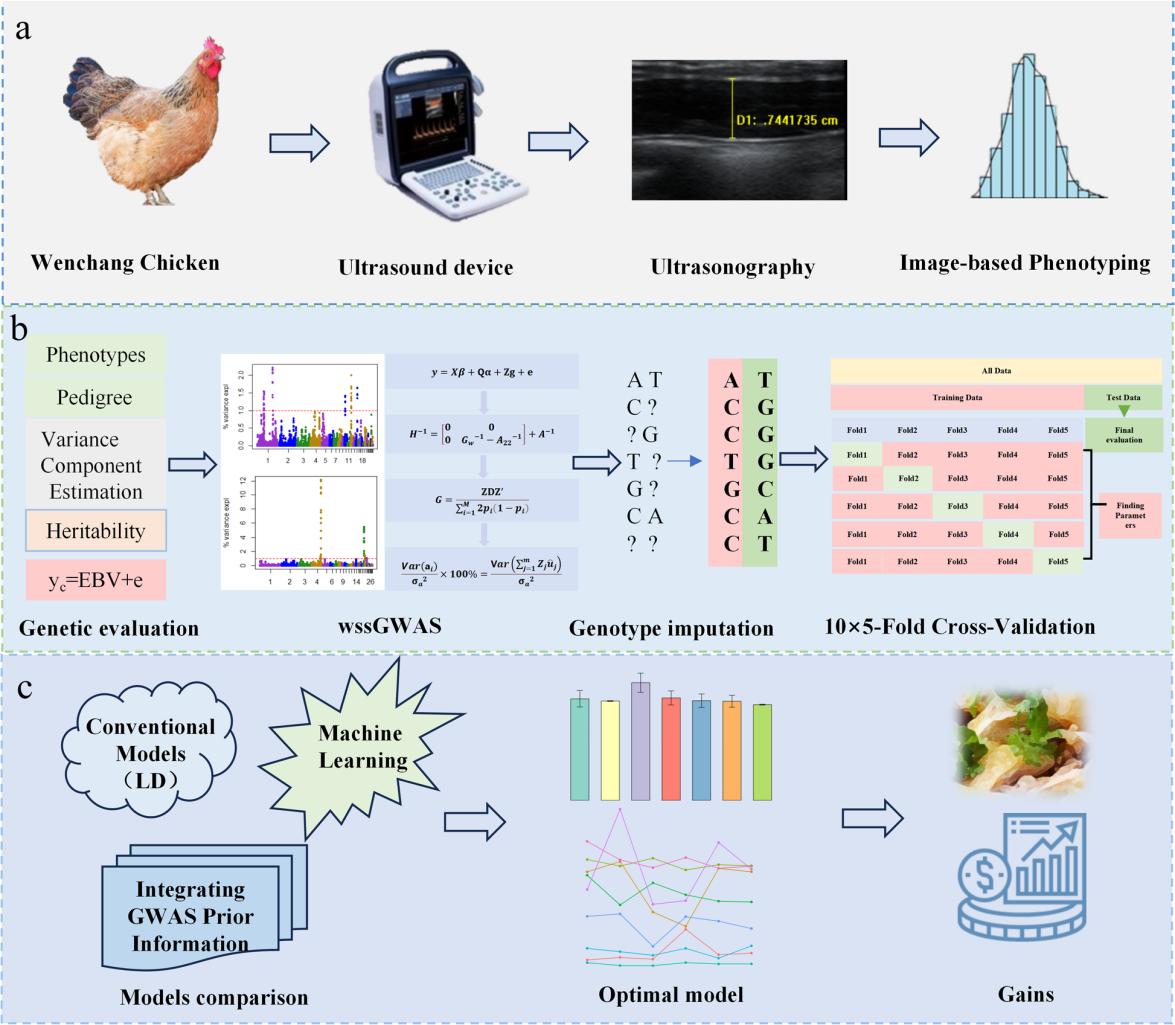
An integrated framework for precision breeding of chicken abdominal fat traits by combining ultrasound phenotyping and multi‑omics prediction

This study established a precision breeding system for chicken abdominal fat traits by integrating ultrasound imaging and multi-omics data (Fig. 1). First, high-throughput phenotyping and standardization of abdominal fat thickness were achieved through multi-stage dynamic ultrasound monitoring (Fig. 1a). Second, by combining SNP arrays, whole-genome sequencing, and multiple prediction models, we developed an integrated genotype–phenotype analysis pipeline encompassing GBLUP, WGBLUP, and machine learning algorithms (Fig. 1b). Finally, the optimal prediction model was selected through repeated cross-validation and applied to breeding practice, thereby completing a closed-loop technical pathway from accurate phenotyping to efficient genomic selection (Fig. 1c). This system provides a scalable methodological framework for genetic improvement of fat-related traits in poultry.

### Phenotypic variation and genetic parameters underpin predictive challenges

**Fig. 2:**
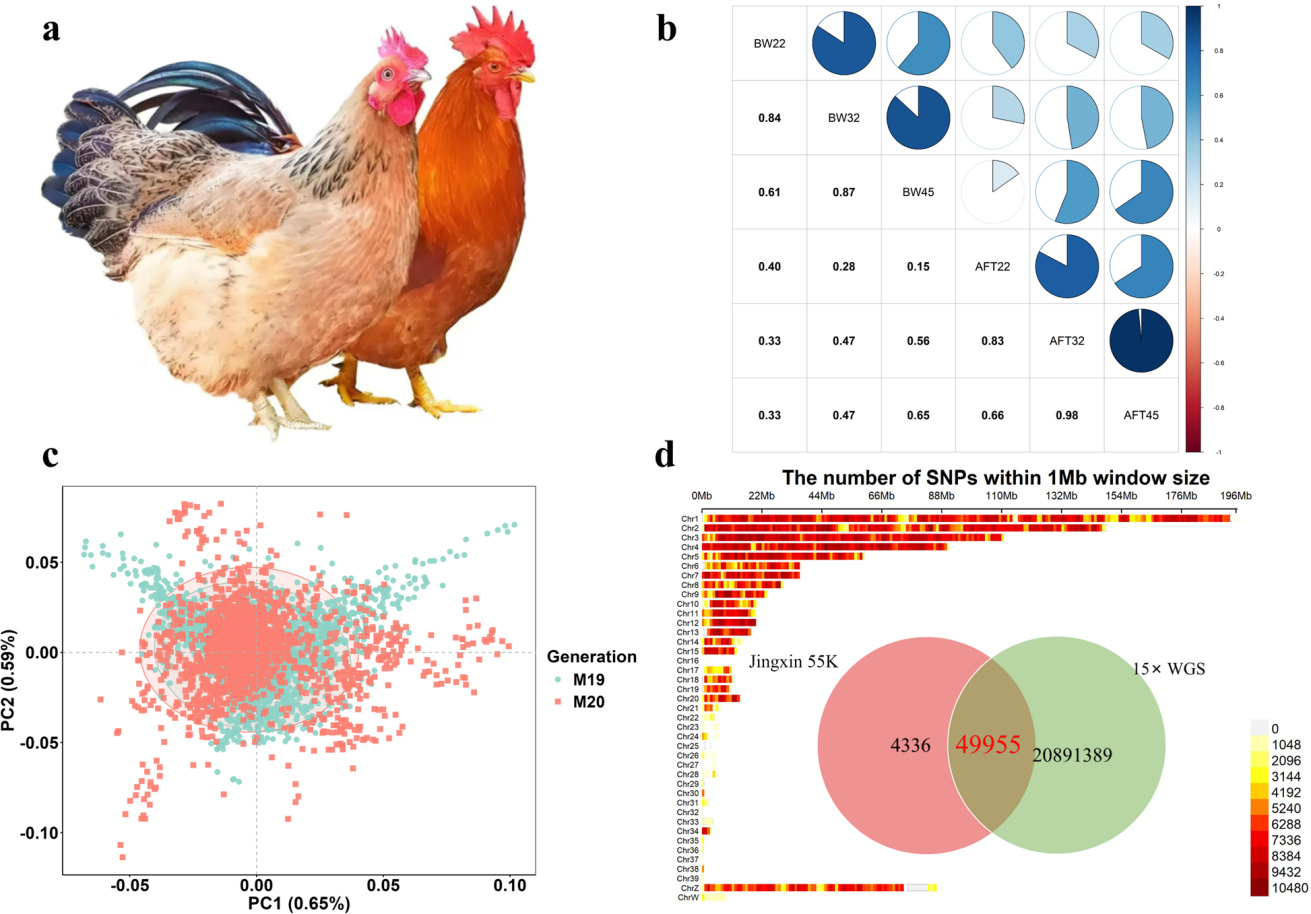
Population overview and genomic characteristics of Wenchang chicken. **a** Images of male and female Wenchang chickens. **b** Visualization of genetic correlation matrix among production traits. Traits abbreviations are displayed on the diagonal, the lower triangle presents numerical values of genetic correlations, while the upper triangle uses sector size to visually represent the correlation strength. **c** PCA plot of the studied population. Ellipses represent 95% confidence intervals for each generation (M19: 19^th^ generation; M20: 20^th^ generation). **d** SNP density distribution on chromosomes after imputation to whole-genome sequencing level, along with a Venn diagram illustrating the overlap between imputed and chip SNP loci

Prior to genomic prediction, descriptive statistics were computed for the target traits. BW increased progressively from 22 to 45 weeks of age, with mean values of 1.774 kg, 1.905 kg, and 1.969 kg, respectively. In contrast, AFT showed a fluctuating trend, declining initially from 0.750 cm at 22 weeks to 0.698 cm at 32 weeks, before rising to 0.762 cm at 45 weeks. Considerable phenotypic variation was observed, as reflected by standard deviations ranging from 0.172 to 0.276 for BW and from 0.210 to 0.234 for AFT. Heritability estimates (with standard errors) for BW across the three ages were 0.155 (0.010), 0.264 (0.012), and 0.200 (0.013), while those for AFT were 0.175 (0.012), 0.254 (0.012), and 0.174 (0.015), with the highest heritability observed at 32 weeks for both traits. Strong pairwise genetic correlations were observed for BW (0.84–0.87) and AFT (0.83–0.98) across the three measurement ages (22, 32, and 45 weeks). Concurrently, moderate positive genetic correlations were evident between BW and AFT at different developmental stages (Fig. 2b).

To evaluate the stability of population genetic structure under continuous artificial selection, principal component analysis (PCA) was conducted on the 19^th^ (M19) and 20^th^ (M20) generations of the Wenchang chicken M‑line. Based on 40,988 SNP markers, the first two principal components explained 0.65% and 0.59% of the total genetic variation, respectively (Fig. 2c). The two consecutive generations showed high overlap in the PC1–PC2 space, with 95% confidence ellipses nearly fully overlapping, indicating that population genetic structure remained stable under ongoing selection and that genetic drift was limited between generations. These results provide a favorable and consistent genetic background for implementing cross‑generational genomic selection in this chicken line. Following imputation of the 55K SNP array data to whole-genome sequence level, a total of 41,195 SNPs common to both platforms were retained. The distribution of SNP density across chromosomes and the overlap of shared loci are visualized in Fig. 2d.

A slaughter validation on 320 chickens confirmed the accuracy of ultrasound-derived AFT. Strong correlations were observed with post-slaughter abdominal fat weight (*r* = 0.760, *P* < 0.001) and percentage (*r* = 0.764, *P* < 0.001) (Fig. S1A and C). Bland–Altman analysis showed excellent agreement, with 94.4% (fat weight) and 95.0% (fat percentage) of points within the 95% limits of agreement and no systematic bias (Fig. S1B and D). These results validate ultrasound as a reliable in vivo phenotyping tool for abdominal fat in Wenchang chickens.

### Incorporating prior information enhances genomic prediction

**Fig. 3:**
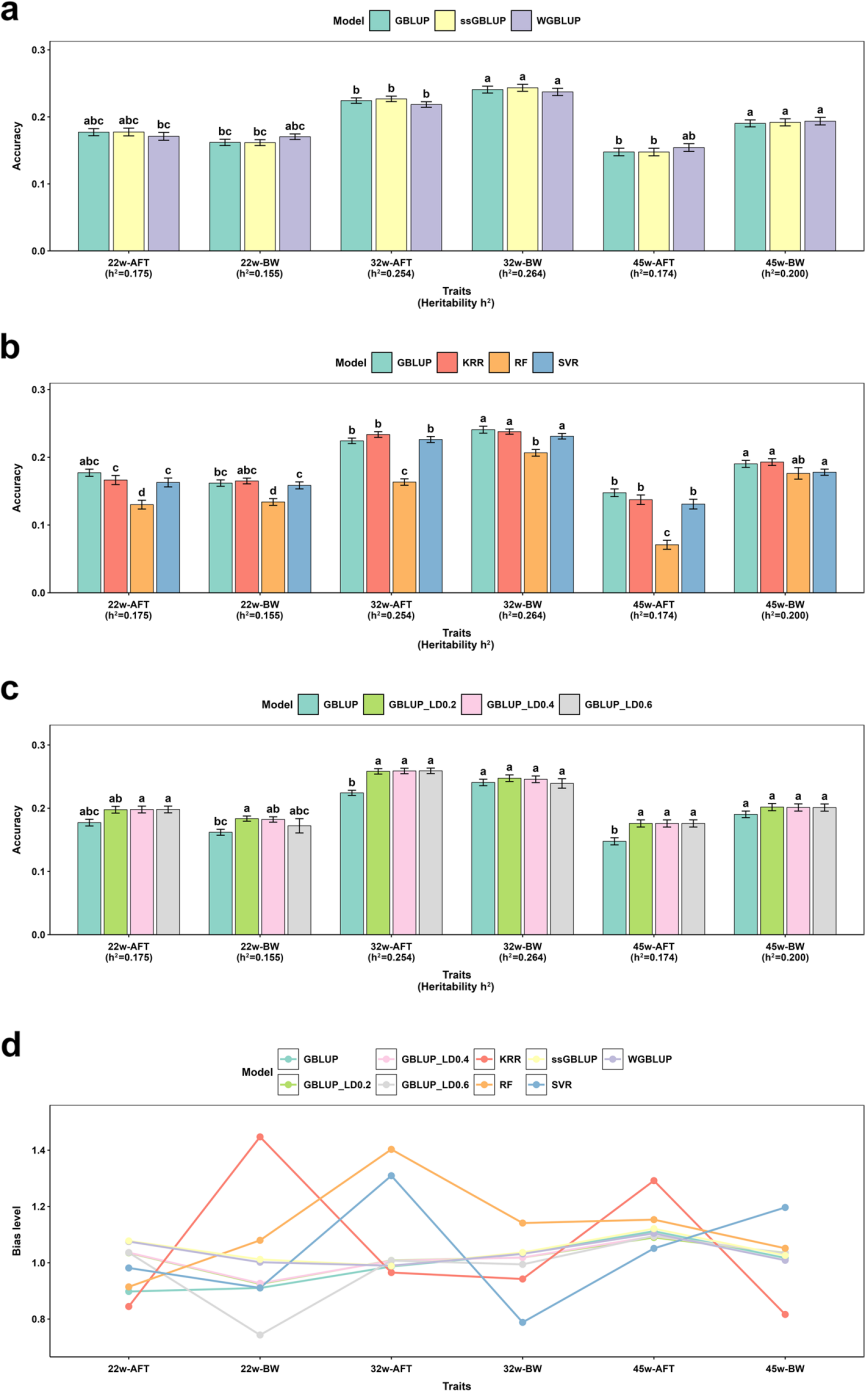
Comprehensive evaluation of genomic prediction models for body weight and abdominal fat thickness. The figure compares the prediction accuracy and bias level of nine genomic prediction methods for body weight (BW) and abdominal fat thickness (AFT) based on 10 repetitions of fivefold cross‑validation. **a** Prediction accuracy of GBLUP, ssGBLUP, and WGBLUP across different traits. **b** Comparative prediction accuracy of GBLUP, SVR, KRR, and RF for each trait. **c** Accuracy of GBLUP under three LD‑pruning thresholds (LD = 0.2, 0.4, 0.6). **d** Comparison of prediction bias among the nine methods for BW and AFT, where values on the *y*-axis closer to 1 indicate better bias level. For panels **a**–**c**, the numerical values above each bar represent the corresponding prediction accuracy, and the error bars denote the standard error. Different lowercase letters above bars indicate significant differences based on Tukey’s HSD test (*P* < 0.05) following one‑way ANOVA of 10 replicated fivefold cross‑validation runs. GBLUP Genomic: Best Linear Unbiased Prediction; ssGBLUP: Single-Step Genomic Best Linear Unbiased Prediction; WGBLUP: Weighted Genomic Best Linear Unbiased Prediction; GBLUP_LD0.2, GBLUP_LD0.4, GBLUP_LD0.6: GBLUP models fitted using SNP subsets pruned at linkage disequilibrium thresholds of *r*^2^ < 0.2, 0.4, and 0.6, respectively. KRR: Kernel Ridge Regression; SVR: Support Vector Regression; RF: Random Forest; LightGBM: Light Gradient Boosting Machine; AdaBoost.R2: AdaBoost for Regression

Figure 3a illustrates the prediction accuracies obtained from ten replicates of five-fold cross-validation for the GBLUP, ssGBLUP, and WGBLUP models, where WGBLUP incorporates SNP weights derived from wssGWAS results. For body weight traits, the GBLUP model based on the 55K SNP chip yielded prediction accuracies of 0.1619, 0.2407, and 0.1903 at 22, 32, and 45 weeks of age, respectively. Overall, WGBLUP—which incorporates prior weights from wssGWAS—consistently achieved the highest accuracy for both body weight and abdominal fat thickness, with average improvements of 2.18% and 0.55%, respectively, over GBLUP. The ssGBLUP model also consistently outperformed GBLUP. Notably, the advantage of WGBLUP was most pronounced at specific time points: it improved prediction accuracy by 5.25% for body weight at 22 weeks and by 4.47% for abdominal fat thickness at 45 weeks. These findings underscore that integrating prior biological information enhances genomic prediction, particularly for traits under strong selection or stage-specific regulation.

#### Machine learning models offer targeted gains for complex traits

Figure 3b shows the average prediction accuracies of various ML methods and GBLUP for BW and AFT traits, based on ten replicates of five-fold cross-validation. Using default parameters, only SVR demonstrated a modest improvement over GBLUP for the 32-week AFT trait, while the other models showed no enhancement. After hyperparameter tuning, KRR achieved the first significant gains, followed by SVR; in contrast, RF failed to improve prediction accuracy under any tested conditions. Specifically, relative to GBLUP, KRR increased the average prediction accuracy for BW by 1.92%, 3.00%, and 1.37% across different time points. SVR exhibited a modest improvement of approximately 0.20% for BW at the first two time points. For AFT measured at three time points, KRR enhanced prediction accuracy by 0.68%, 4.15%, and 0.20%, while SVR improved accuracy by only 2.1% at 32 weeks. Among the ML approaches, RF consistently performed the poorest for the traits evaluated. The optimal hyperparameters for KRR, SVR, and RF, determined via grid search, are summarized in Table 2. Additionally, ensemble learning methods AdaBoost.R2 and LightGBM were assessed. Despite their theoretical advantages in modeling nonlinear relationships, these methods generally yielded lower prediction accuracies than GBLUP and other ML models in this study (detailed results are provided in Table S1) and thus are not discussed in detail in the main text.

**Table 2:** The optimal hyperparameters of each ML model obtained through a grid search for BW and AFT traits in 10 replicates of fivefold CV

Model^1^	Optimal hyperparameters^2^	
	**BW**	**AFT**
KRR	kernel = ‘rbf’, λ = 0.1, gamma = 0.0001	
SVR	kernel = ‘rbf’, C = 1.0, gamma = 0.0001	kernel = ‘rbf’, C = 0.1, gamma = 0.0001
RF	n_estimators = 200, max_depth = 10, min_samples_split = 5	
LightGBM	learning_rate = 0.01, max_depth = 5, n_estimators = 200, num_leaves = 15	
AdaBoost.R2	learning_rate = 0.01, n_estimators = 100	learning_rate = 0.01, n_estimators = 50

^1^KRR: Kernel Ridge Regression; SVR: Support Vector Regression; RF: Random Forest; LightGBM: Light Gradient Boosting Machine; AdaBoost.R2: AdaBoost for Regression

^2^Optimal hyperparameters: The optimal hyperparameters of each machine learning method obtained by using a grid search

#### Optimal marker density is trait-specific, reflecting underlying genetic architecture

Figure 3c presents bar plots of prediction accuracies from five-fold cross-validation repeated ten times under varying SNP marker densities. The compared models include the original GBLUP based on the 55K SNP chip and three GBLUP_LD models derived from WGS-imputed genotypes pruned at different LD thresholds (LD0.2, LD0.4, and LD0.6). For body weight traits, the prediction accuracies of GBLUP_LD0.2 at 22, 32, and 45 weeks were 0.1835, 0.2474, and 0.2017, respectively. The respective prediction accuracies for GBLUP_LD0.4 were 0.1822, 0.2457, and 0.2012, while those for GBLUP_LD0.6 were 0.1722, 0.2442, and 0.2010, respectively. GBLUP_LD0.2 consistently achieved the highest prediction accuracy across all time points, suggesting that this LD threshold offers an optimal marker density for genomic prediction of body weight. On average, it improved prediction accuracy by 6.58% compared to the original GBLUP model. For abdominal fat thickness, prediction accuracies at 22 weeks were 0.1976, 0.1979, and 0.1980 for GBLUP_LD0.2, GBLUP_LD0.4, and GBLUP_LD0.6, respectively; at 32 weeks, they were 0.2583, 0.2589, and 0.2591; and at 45 weeks, all three models produced identical values of 0.1759. GBLUP_LD0.6 exhibited the best overall performance across the three stages, with an average improvement of 15.30% over the original GBLUP. These findings highlight that appropriate LD pruning of WGS-level SNP data can optimize marker distribution and substantially enhance the accuracy of genomic predictions. Moreover, genetic evaluations using SNPs obtained after pruning from WGS data resulted in improved estimated heritability for all traits. Detailed heritability values for each trait can be found in Table S2.

### Bias level

The regression coefficient of *y*_c_ on predicted values (PV) was used to evaluate the prediction bias level of each model. As shown in Fig. 3d, the average bias level coefficients for GBLUP across three time points were 0.9863 for BW and 0.9989 for AFT, indicating no evidence of dispersion bias. For BW traits, WGBLUP (1.0142) and ssGBLUP (1.0228) showed bias level closer to the ideal value of 1 compared to GBLUP, whereas the opposite trend was observed for AFT traits. Among GBLUP models with different LD pruning thresholds (LD0.2, LD0.4, LD0.6), the prediction bias level for BW traits initially improved and then declined, with GBLUP_LD0.4 performing best and surpassing the standard GBLUP model. For AFT traits, GBLUP_LD0.2 exhibited bias level closer to 1 but did not exceed GBLUP. ML models such as KRR, SVR, and RF demonstrated relatively poor bias level with default parameters. After hyperparameter tuning via grid search, their bias level approached 1; however, RF and KRR still underperformed relative to GBLUP, while only SVR outperformed GBLUP in bias level. Overall, the WGBLUP model showed the closest bias level to 1 for 22-week BW (1.0019), whereas GBLUP_LD0.4 was closest to 1 for 32-week AFT (1.0071).

### MSE and MAE

**Fig. 4:**
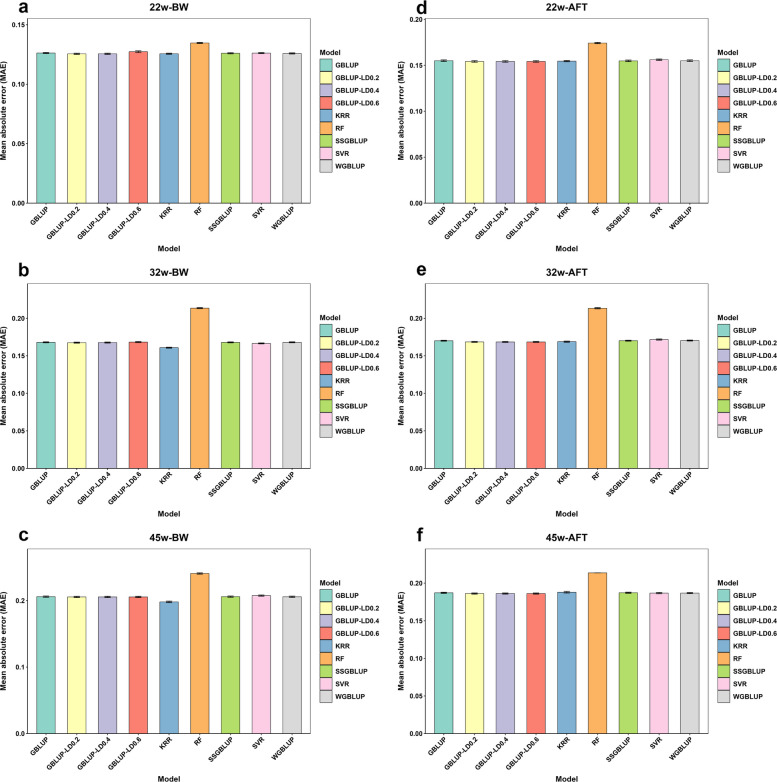
Comparison of mean absolute error across models for body weight and abdominal fat thickness at three time points. **a–c** Body weight (BW) traits measured at 22, 32, and 45 weeks of age respectively. **d–f** Abdominal fat thickness (AFT) traits at the corresponding time points. Each panel presents the average mean absolute error (MAE) derived from ten replicates of five-fold cross-validation across various prediction models. Error bars indicate the standard error. Model: GBLUP Genomic: Best Linear Unbiased Prediction; ssGBLUP: Single-Step Genomic Best Linear Unbiased Prediction; WGBLUP: Weighted Genomic Best Linear Unbiased Prediction; GBLUP_LD0.2, GBLUP_LD0.4, GBLUP_LD0.6: GBLUP models fitted using SNP subsets pruned at linkage disequilibrium thresholds of *r*^2^ < 0.2, 0.4, and 0.6, respectively. KRR: Kernel Ridge Regression; SVR: Support Vector Regression; RF: Random Forest; LightGBM: Light Gradient Boosting Machine; AdaBoost.R2: AdaBoost for Regression

Model performance was further evaluated using MSE and MAE. As presented in Table 3 and Fig. 4, both WGBLUP and ssGBLUP consistently outperformed GBLUP in terms of lower MAE and MSE across all evaluated traits. For instance, for 22-week BW, GBLUP yielded higher prediction errors (MAE: 0.1262; MSE: 0.0254) compared to WGBLUP (MAE: 0.1258; MSE: 0.0252) and ssGBLUP (MAE: 0.1260; MSE: 0.0253). Additionally, for BW traits, the MSE and MAE performances of GBLUP_LD0.2, GBLUP_LD0.4, and GBLUP_LD0.6 followed their prediction accuracy trends, with GBLUP_LD0.2 achieving the lowest MAE values (0.1255 ± 0.0005, 0.1675 ± 0.0007, and 0.2055 ± 0.0010) and MSE values (0.0251 ± 0.0002, 0.0450 ± 0.0004, and 0.0684 ± 0.0007). However, these three models performed similarly for AFT traits. ML methods with default parameters exhibited higher MAE and MSE than GBLUP; following hyperparameter optimization, KRR and SVR generally outperformed GBLUP on these metrics and performed comparably to ssGBLUP and WGBLUP overall. Among ML methods, KRR produced the lowest average MSE (0.0397 for BW; 0.0470 for AFT) and MAE (0.1615 for BW; 0.1704 for AFT) across time points, whereas RF performed the worst, with MSEs of 0.0471 (BW) and 0.0478 (AFT), and MAEs of 0.2004 (BW) and 0.1963 (AFT). Taken together, among the nine methods evaluated, those based on WGS-imputed data combined with LD pruning consistently achieved lower MAE and MSE values across all traits. Table 3 presents MSE values as means with corresponding standard errors (mean ± SE).

**Table 3:** Mean squared error (MSE) of nine methods for BW and AFT as assessed with 10 replicates of fivefold CV (mean ± SE)

Model^1^	22w-BW	32w-BW	45w-BW	22w-AFT	32w-AFT	45w-AFT
GBLUP	0.0254 ± 0.0002	0.0452 ± 0.0004	0.0687 ± 0.0007	0.0376 ± 0.0003	0.0475 ± 0.0004	0.0563 ± 0.0006
SSGBLUP	0.0253 ± 0.0002	0.0452 ± 0.0004	0.0687 ± 0.0007	0.0375 ± 0.0004	0.0475 ± 0.0004	0.0563 ± 0.0006
WGBLUP	0.0252 ± 0.0002	0.0452 ± 0.0004	0.0686 ± 0.0007	0.0376 ± 0.0004	0.0477 ± 0.0004	0.0562 ± 0.0006
GBLUP_LD0.2	0.0251 ± 0.0002	0.0450 ± 0.0004	0.0684 ± 0.0007	0.0372 ± 0.0004	0.0467 ± 0.0004	0.0558 ± 0.0006
GBLUP_LD0.4	0.0251 ± 0.0002	0.0451 ± 0.0004	0.0684 ± 0.0007	0.0372 ± 0.0004	0.0467 ± 0.0004	0.0558 ± 0.0006
GBLUP_LD0.6	0.0259 ± 0.0003	0.0454 ± 0.0004	0.0684 ± 0.0007	0.0372 ± 0.0004	0.0467 ± 0.0004	0.0558 ± 0.0006
KRR	0.0253 ± 0.0002	0.0451 ± 0.0003	0.0686 ± 0.0006	0.0375 ± 0.0003	0.0474 ± 0.0004	0.0561 ± 0.0009
SVR	0.0254 ± 0.0002	0.0453 ± 0.0003	0.0689 ± 0.0006	0.0375 ± 0.0003	0.0479 ± 0.0005	0.0570 ± 0.0009
RF	0.0256 ± 0.0002	0.0456 ± 0.0003	0.0701 ± 0.0007	0.0379 ± 0.0003	0.0478 ± 0.0004	0.0576 ± 0.0009

^1^GBLUP Genomic: Best Linear Unbiased Prediction; ssGBLUP: Single-Step Genomic Best Linear Unbiased Prediction; WGBLUP: Weighted Genomic Best Linear Unbiased Prediction; GBLUP_LD0.2, GBLUP_LD0.4, GBLUP_LD0.6: GBLUP models fitted using SNP subsets pruned at linkage disequilibrium thresholds of *r*^*2*^ < 0.2, 0.4, and 0.6, respectively. KRR: Kernel Ridge Regression; SVR: Support Vector Regression; RF: Random Forest; LightGBM: Light Gradient Boosting Machine; AdaBoost.R2: AdaBoost for Regression

## Computing time

Table 4 summarizes the average computational time per fold for each genomic prediction method during CV in this study. The analyses were conducted on a high-performance computing cluster running Rocky Linux 5.6, consisting of two types of compute nodes: c01/c02 series CPU nodes, each equipped with dual Intel Xeon Platinum 8458P processors (2.7 GHz base frequency). The cluster includes 640 nodes with 1 TB DDR4 RAM and 396 nodes with 512 GB DDR4 RAM. Computation times for all methods are reported in minutes. Among the methods tested, the scikit-learn-based KRR exhibited the highest computational efficiency, requiring only 0.02 to 0.04 min per fold on average. This was substantially faster than the mixed-model methods implemented in BLUPF90: GBLUP (1.27–1.91 min), WGBLUP (1.54–2.05 min), and ssGBLUP (1.62–2.04 min). SVR demonstrated moderate efficiency, with runtimes between 0.39 and 1.21 min, outperforming the GBLUP variants but slower than KRR. RF, however, was the slowest among the machine learning methods, with fold times ranging from 22.74 to 65.78 min. Additionally, GBLUP models based on WGS-imputed SNPs with linkage disequilibrium (LD) pruning showed computation times increasing with marker density: GBLUP_LD0.2 took 7.56–7.85 min, GBLUP_LD0.4 required 19.13–20.74 min, and GBLUP_LD0.6 demanded 20.80–32.52 min per fold. Importantly, the differences in computation time are attributable not just to the algorithms, but also to a major factor: the efficiency of their software implementations (BLUPF90 vs. scikit-learn).

**Table 4:** Average computing time to complete each fold of fivefold CV according to different genomic prediction methods

Model^1^	22w-BW	32w-BW	45w-BW	22w-AFT	32w-AFT	45w-AFT
GBLUP	1.43 min	1.27 min	1.44 min	1.67 min	1.36 min	1.91 min
SSGBLUP	2.04 min	1.64 min	1.62 min	1.69 min	1.63 min	1.80 min
WGBLUP	1.54 min	1.72 min	1.64 min	2.05 min	1.70 min	1.79 min
GBLUP_LD0.2	7.76 min	7.56 min	7.57 min	7.77 min	7.60 min	7.85 min
GBLUP_LD0.4	20.74 min	19.13 min	20.28 min	20.74 min	19.13 min	20.28 min
GBLUP_LD0.6	20.80 min	32.49 min	31.86 min	31.66 min	31.64 min	32.52 min
KRR	0.04 min	0.04 min	0.03 min	0.02 min	0.03 min	0.02 min
SVR	1.06 min	1.21 min	0.74 min	0.74 min	1.05 min	0.39 min
RF	1 h 6 min	30.27 min	45.78 min	22.74 min	37.45 min	31.81 min

^1^GBLUP Genomic: Best Linear Unbiased Prediction; ssGBLUP: Single-Step Genomic Best Linear Unbiased Prediction; WGBLUP: Weighted Genomic Best Linear Unbiased Prediction; GBLUP_LD0.2, GBLUP_LD0.4, GBLUP_LD0.6: GBLUP models fitted using SNP subsets pruned at linkage disequilibrium thresholds of r^2^ < 0.2, 0.4, and 0.6, respectively. KRR: Kernel Ridge Regression; SVR: Support Vector Regression; RF: Random Forest; LightGBM: Light Gradient Boosting Machine; AdaBoost.R2: AdaBoost for Regression

## Discussion

Our study delivers two pivotal contributions to poultry genomics: the establishment of ultrasound imaging as a scalable platform for in vivo phenotyping, and a systematic dissection of how genetic architecture dictates the performance of genomic prediction models. The moderate heritability observed for both BW and AFT, peaking at 32 weeks of age, underscores their genetic basis and highlights a critical developmental window for selection. This pattern is consistent with previous reports [33, 34], suggesting that this time point may represent a critical window for implementing effective selection strategies. The novel application of non-invasive ultrasound measurement of AFT in poultry successfully transitions a traditionally postmortem trait into a longitudinal, animal welfare phenotype, thereby overcoming a major bottleneck in breeding for carcass composition traits. While our ultrasound-based phenotyping protocol proved practical and efficient, potential operator or device effects were not modeled statistically due to lack of systematic metadata tracking during high-throughput data collection. Future applications should implement standardized recording of operator and device information. Including these factors as covariates in sensitivity analyses—especially in multi-operator or multi-center breeding programs—would strengthen the robustness and generalizability of genomic predictions using ultrasound-derived traits.

The consistent superiority of ssGBLUP over traditional GBLUP reaffirms the value of integrating pedigree and genomic relationships, thereby enhancing the accuracy of genetic evaluations through a comprehensive representation of the genetic architecture[1, 11, 35, 36]. To further improve predictive performance, we implemented the WGBLUP. Unlike the traditional GBLUP model, which assumes equal contributions of all SNPs to the genetic variance [35], WGBLUP assigns differential weights to individual SNPs based on their estimated associations with the target traits [27], better capturing the underlying genetic complexity. We posit that this improvement stems from the model's ability to recalibrate the genomic relationship matrix by upweighting SNPs in or near QTL regions identified in our prior wssGWAS [34]. These findings are further supported by multiple independent studies, which have confirmed the effectiveness of incorporating functional information or association-based weights into genomic prediction models [36, 37].This mechanism allows WGBLUP to transition from assuming a purely polygenic background to approximating a mixed architecture of large and small-effect loci. Overall, our results support the broader application of WGBLUP as a promising strategy for enhancing the accuracy of genetic evaluations in poultry and other livestock species.

A fundamental assumption of SNP chip-based genomic selection is that each quantitative trait locus (QTL) is in LD with at least one nearby SNP marker [38, 39]. However, due to the limited density of commercial SNP arrays, some functional loci—particularly those with low minor allele frequencies or rare mutations—may not be adequately captured. This limitation can compromise the effectiveness of genomic prediction, especially for complex traits influenced by many small-effect variants. Conversely, WGS theoretically enables the detection of nearly all functional mutations across the genome, thereby reducing the reliance on LD and improving the resolution and accuracy of genetic evaluations [38, 39]. To balance predictive performance with economic feasibility, this study employed genotype imputation to elevate the marker density of SNP chip data to a WGS-like level. We then applied LD pruning to construct SNP panels of varying densities based on different LD thresholds. A pronounced contrast exists between the genetic foundations of BW and AFT, a finding consistently upheld by SNP panel analyses and our team's earlier wssGWAS [34]. For BW, the combination of accurate prediction from a sparse LD0.2 panel and a high proportion of genetic variance (19.05%) explained by the top three QTLs strongly suggests an architecture shaped by moderate-effect loci. For AFT, however, the congruent evidence—the need for a dense LD0.6 panel for optimal prediction and the low variance (5.87%) explained by the top QTLs—converge to support a highly polygenic model driven by the cumulative contribution of numerous small-effect variants. These observations are consistent with previous findings [7], reinforcing the idea that the integration of WGS-imputed genotypes with LD pruning offers a powerful and flexible strategy [10, 40] for improving genomic prediction accuracy in poultry. Such approaches not only enhance the capture of functional variation but also allow for customized marker panels tailored to specific traits and populations, supporting more efficient and cost-effective genomic selection in breeding programs.

Beyond this, this study systematically evaluated the performance of several mainstream ML methods in genomic prediction of body weight and abdominal fat thickness traits in chickens, and compared them with the conventional GBLUP model. The results showed that SVR and KRR achieved improvements of 4.15% and 2.10%, respectively, in predictive accuracy for abdominal fat thickness at 32 weeks of age, demonstrating clear advantages. Compared to GBLUP, ML methods are better suited to handle high-dimensional data with relatively small sample sizes [41], and they do not rely on assumptions of additive genetic architecture. This allows them to flexibly capture potential nonlinear relationships between genotypes and phenotypes, particularly in the presence of non-additive effects such as epistasis. However, for traits predominantly governed by additive effects, linear models like GBLUP often demonstrate greater stability and reliability [39, 42]. These findings are consistent with previous studies. For instance, research in cattle, pigs, and crops has shown that ML approaches may outperform linear models for certain complex traits or under low LD conditions, while GBLUP remains more stable for traits primarily influenced by additive effects [43–45]. One study reported that the prediction accuracy of GBLUP for birth weight and yearling weight in cattle was significantly higher (by 10%–15%) than that of ML models such as RF, SVM, and Convolutional Neural Network (CNN), although RF outperformed GBLUP slightly for weaning weight after fine-tuning [43]. In pigs, Su et al. [44] found that different ML algorithms and GBLUP each had advantages for specific traits, with SVR, KRR, and ensemble methods like Stacking performing well across multiple traits. Similarly, Farooq et al. [45] reported that ML models outperformed rBLUP for disease resistance traits but showed less consistent performance for traits with simple additive architectures.

Notably, although some studies (e.g., Liang et al. [46]) have reported superior performance of ensemble methods such as AdaBoost in other species, Adaboost.R2 and LightGBM did not outperform GBLUP or the leading ML models (e.g., SVR and KRR) for the traits analyzed in this study (see Table S1). Several factors may account for this: (1) the traits studied are mainly controlled by additive genetic effects, limiting the advantage of boosting-based models; (2) the tuning process for Adaboost.R2 is complex, and full optimization was not feasible under current computational resources; and (3) LightGBM can be sensitive to hyperparameters in high-dimensional SNP datasets, increasing the risk of underfitting. Additionally, the performance of RF was likely affected by constraints in parameter tuning and computational efficiency. Taken together, the performance of ML methods further corroborates this architecture-dependent pattern. The significant gains from SVR and KRR for AFT, a trait governed by complex physiology, suggest their capacity to model subtle non-additive relationships that linear models may miss. Conversely, the robust performance of linear models for BW underscores the continued dominance of additive effects for this trait. The failure of ensemble methods like RF and LightGBM likely results from their susceptibility to overfitting in high-dimensional SNP space without exceedingly large sample sizes, a known challenge in genomic ML.

Collectively, our findings advocate for a trait-aware genomic prediction framework. For traits with evidence of major QTLs, WGBLUP is highly effective. For highly polygenic complex traits, leveraging WGS data with relaxed LD pruning or non-linear ML models like KRR can yield significant gains. For standard additive traits, the computational simplicity of (ss)GBLUP remains compelling. This framework moves beyond one-size-fits-all solutions, guiding breeders to strategically match the analytical tool to the biological question.

Finally, while GS holds immense promise, its cost-effectiveness is crucial for widespread adoption. The combination of non-invasive ultrasound phenotyping with cost-efficient genotype imputation and tailored model selection, as demonstrated here, provides a practical and powerful pathway to accelerate genetic gain for complex traits in poultry breeding. Future efforts should focus on refining this trait-aware framework. The high-density genotypic data generated in this study provides a critical platform for the next phase: linking pivotal genomic signals to candidate genes and functional pathways to deepen mechanistic understanding. Building on this biological foundation, the integration of multi-omics data and the development of pre-hoc metrics to predict trait architecture will ultimately enable fully informed and dynamic genomic selection strategies.

## Conclusions

Our study confirms that optimal genomic prediction strategies for Wenchang chickens are trait-specific, dictated by the underlying genetic architecture. We overcame a key barrier in carcass trait breeding by implementing ultrasound imaging as a scalable, non-invasive phenotyping platform. Our comprehensive analysis validated two superior approaches for enhancing prediction of complex traits like abdominal fat thickness: integrating prior biological knowledge via WGBLUP (informed by wssGWAS) and refining marker density from WGS data through LD pruning, both of which substantially outperformed standard GBLUP. Computationally efficient machine learning models, notably KRR and SVR, further delivered competitive accuracy with operational speed. Collectively, we provide a trait-informed framework that empowers breeders to maximize genetic gain efficiently in poultry breeding programs.

## Supplementary Information

Additional file 1: Fig. S1. Validation of ultrasound-based abdominal fat thickness (AFT) measurement. (A) Scatter plot showing the correlation between ultrasound AFT and slaughter-measured abdominal fat weight (AFW). (B) Bland-Altman plot assessing agreement between ultrasound and slaughter measurements for AFW. (C) Scatter plot showing correlation between ultrasound AFT and abdominal fat percentage (AFP). (D) Bland-Altman plot assessing agreement for AFP. Solid lines represent regression lines (A, C) or mean differences (B, D); dashed lines indicate 95% limits of agreement. Statistical parameters including Pearson's correlation coefficient (*r*), coefficient of determination (*R*²), and mean bias are displayed on each panel.

Additional file 2. Detailed methodologies of LightGBM and AdaBoost.R2 models.

Additional file 3: Table S1. Genomic prediction performance of LightGBM and AdaBoost.R2 for BW and AFT at different growth stages based on 10 repeats of 5-fold cross-validation.

Additional file 4: Table S2. Summary of genetic evaluations using LD-pruned WGS-level SNPs.

## Data Availability

The datasets used or analyzed during the present study are available from the corresponding author on reasonable request.

## Abbreviations

AdaBoost.R2: Adaptive boosting for regression
CV: Cross-validation
DR2: Dosage R-squared measure
EBV: Estimated breeding values
GBLUP: Genomic best linear unbiased prediction
GEBV: Genomic estimated breeding value
GP: Genomic prediction
GS: Genomic selection
GWAS: Genome-wide association study
KRR: Kernel ridge regression
LD: Linkage disequilibrium
LightGBM: Light gradient boosting machine
MAE: Mean absolute error
ML: Machine learning
MSE: Mean squared error
RF: Random forest
SNP: Single nucleotide polymorphism
ssGBLUP: Single-step genomic best linear unbiased prediction
SVR: Support vector regression
WGBLUP: Weighted genomic best linear unbiased prediction
WGS: Whole genome sequence
wssGWAS: Weighted single-step genome-wide association study
*y*_c_: Corrected phenotypes
