# Supplementary figures and images for: Enhancing genomic prediction for key production traits in chickens through ultrasound phenotyping and multi-model comparative analysis
Source: J Anim Sci Biotechnol. 2026 Apr 25;17:78. doi: 10.1186/s40104-026-01384-0 (PMC13109885; doi:10.1186/s40104-026-01384-0)

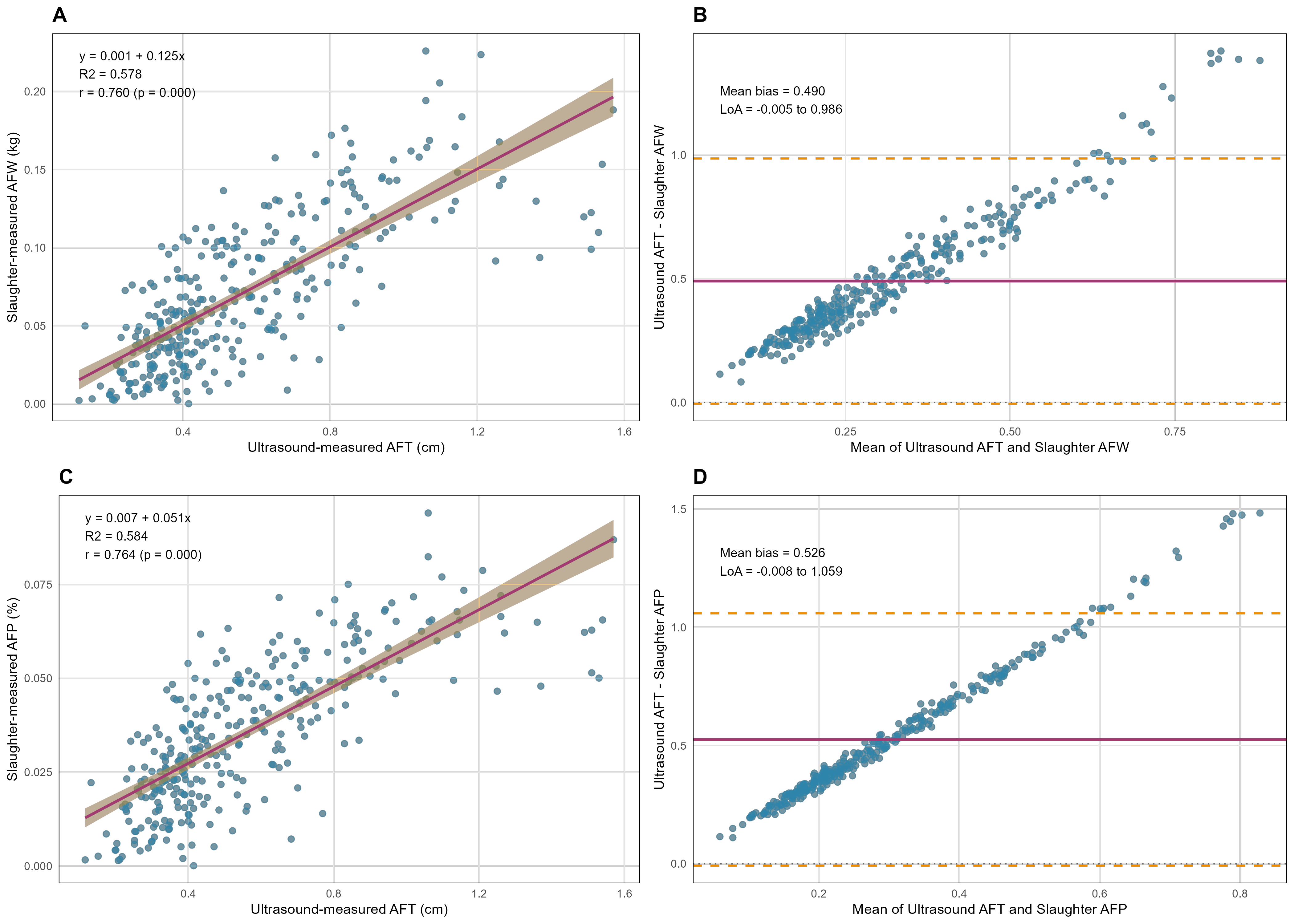

Supplement: Supplementary file 1 — Additional file 1: Fig. S1. Validation of ultrasound-based abdominal fat thickness (AFT) measurement. (A) Scatter plot showing the correlation between ultrasound AFT and slaughter-measured abdominal fat weight (AFW). (B) Bland-Altman plot assessing agreement between ultrasound and slaughter measurements for AFW. (C) Scatter plot showing correlation between ultrasound AFT and abdominal fat percentage (AFP). (D) Bland-Altman plot assessing agreement for AFP. Solid lines represent regression lines (A, C) or mean differences (B, D); dashed lines indicate 95% limits of agreement. Statistical parameters including Pearson's correlation coefficient (r), coefficient of determination (R²), and mean bias are displayed on each panel. [file 40104_2026_1384_MOESM1_ESM.tiff]
