# Supplementary material for: Enhancing genomic prediction for key production traits in chickens through ultrasound phenotyping and multi-model comparative analysis
Source: J Anim Sci Biotechnol. 2026 Apr 25;17:78. doi: 10.1186/s40104-026-01384-0 (PMC13109885; doi:10.1186/s40104-026-01384-0)
Supplement: Supplementary file 2 — Additional file 2. Detailed methodologies of LightGBM and AdaBoost.R2 models. [file 40104_2026_1384_MOESM2_ESM.docx]

**Light gradient boosting machine**

LightGBM is a highly efficient machine learning framework based on the gradient boosting decision tree (GBDT) algorithm, widely applied in both classification and regression tasks[27]. It constructs an additive model by sequentially training multiple weak learners (i.e., decision trees). The general form of the LightGBM model is as follows:：

**1**

$${\hat{\boldsymbol{y}}}_{\boldsymbol{i}}=\sum_{m=1}^{M} \boldsymbol{f}_{\boldsymbol{m}}\left( \boldsymbol{x}_{\boldsymbol{i}} \right)\mathbf{,}\boldsymbol{f}_{\boldsymbol{m}}\boldsymbol{\epsilon F}$$

where $\hat{y}_{i}$ is the predicted value for sample **X*_i_*​**, $\boldsymbol{f}_{\boldsymbol{m}}$​ represents the m-th weak learner (decision tree), and $\boldsymbol{F}$ denotes the functional space of all possible tree structures, including leaf weights and splitting rules [28].

**Adaptive boosting for regression**AdaBoost.R2 is a regression-oriented extension of the AdaBoost algorithm, designed under the boosting framework to handle continuous outcome prediction [29]. It iteratively trains a series of weak learners (e.g., regression trees), and updates the sample weight distribution based on prediction errors, allowing the model to focus on difficult-to-predict samples and thereby improving overall regression performance. The final output is determined by the weighted median of predictions from all weak learners, and the model can be expressed as:

**2**

$$y\mathbf{=}inf\left[ \boldsymbol{y \epsilon Y:}\sum_{\boldsymbol{t:}\boldsymbol{f}_{\boldsymbol{t}}\boldsymbol{(x)\leq y}} \log\frac{\boldsymbol{1}}{\boldsymbol{\varepsilon}_{\boldsymbol{t}}}\boldsymbol{\geq}\frac{\boldsymbol{1}}{\boldsymbol{2}}\sum_{\boldsymbol{t}} \log\frac{\boldsymbol{1}}{\boldsymbol{\varepsilon}_{\boldsymbol{t}}} \right]$$

where $\boldsymbol{f}_{\boldsymbol{t}}\left( \boldsymbol{x} \right)$ is the prediction of the $t$-th weak learner, and $\boldsymbol{\varepsilon}_{\boldsymbol{t}}$​ denotes the relative error, defined as: $\boldsymbol{\varepsilon}_{\boldsymbol{t}}\boldsymbol{=}{\bar{L}_{t}}/\left( 1-\bar{L}_{t} \right)$， $\bar{L}_{t}=\sum_{i=1}^{m} \mathsf{L}_{\mathsf{t}}(i)\mathsf{D}_{\mathsf{t}}(i)$。$L_{\mathsf{t}}\left( i \right)$is the loss between the observed and predicted values for sample i, and $\mathsf{D}_{\mathsf{t}}\left( i \right)$ is the weight of the i-th sample at iteration t After each iteration, the weights are updated as:

**14**

$$\mathsf{D}_{\mathsf{t+}\boldsymbol{1}}\left( \boldsymbol{i} \right)=\frac{\mathsf{D}_{\mathsf{t}}\boldsymbol{(i)}{\boldsymbol{\beta}_{\boldsymbol{t}}}^{\boldsymbol{(1-}\boldsymbol{L}_{\mathsf{t}}\boldsymbol{(i))}}}{\boldsymbol{Z}_{\boldsymbol{t}}}$$

where $Z_{t}$is a normalization factor ensuring that $\mathsf{D}_{\mathsf{t}+1}\left( i \right)$ forms a valid probability distribution.
