# Supplementary material for: Enhancing genomic prediction for key production traits in chickens through ultrasound phenotyping and multi-model comparative analysis
Source: J Anim Sci Biotechnol. 2026 Apr 25;17:78. doi: 10.1186/s40104-026-01384-0 (PMC13109885; doi:10.1186/s40104-026-01384-0)
Supplement: Supplementary file 4 — Additional file 4: Table S2. Summary of genetic evaluations using LD-pruned WGS-level SNPs. [file 40104_2026_1384_MOESM4_ESM.docx]

| **Traits** | **22w-BW** | **32w-BW** | **45w-BW** | **22w-AFT** | **32w-AFT** | **45w-AFT** |
| --- | --- | --- | --- | --- | --- | --- |
| 𝜎_𝑔_^2^ | 0.005 | 0.015 | 0.017 | 0.008 | 0.016 | 0.014 |
| 𝜎_e_^2^ | 0.021 | 0.034 | 0.055 | 0.030 | 0.035 | 0.044 |
| h^2^(Se) | 0.190(0.006) | 0.306(0.008) | 0.230(0.008) | 0.216(0.008) | 0.312(0.009) | 0.236(0.010) |
